# Appetite Regulation and Allostatic Load Across Prediabetes Phenotypes

**DOI:** 10.3390/nu18010158

**Published:** 2026-01-03

**Authors:** Steven K. Malin, Emily M. Heiston

**Affiliations:** 1 Department of Kinesiology & Health, Rutgers University, New Brunswick, NJ 08901, USA; emily.heiston@rutgers.edu; 2 Division of Endocrinology, Metabolism & Nutrition, Rutgers University, New Brunswick, NJ 08901, USA; 3 New Jersey Institute for Food, Nutrition and Health, Rutgers University, New Brunswick, NJ 08901, USA; 4 Institute of Translational Medicine and Science, Rutgers University, New Brunswick, NJ 08901, USA

**Keywords:** weight regulation, stress, nutrition, insulin sensitivity, appetite hormones

## Abstract

Allostatic load is a physiological measure of chronic stress, and stress is implicated in disrupting appetite regulation. Individuals with obesity and type 2 diabetes have higher allostatic load compared to lean counterparts. However, whether allostatic load differs across prediabetes phenotypes and relates to appetite is unknown. Purpose: Test whether prediabetes phenotypes differ in allostatic load in relation to altered appetite regulation. Methods: Individuals with obesity were recruited, and prediabetes was determined using American Diabetes Association (ADA) criteria (75 g OGTT) for this cross-sectional study. After an overnight fast, appetite hormones (ghrelin and PYY), insulin, and glucose were measured every 30 min up to 120 min of the OGTT. Perception of hunger and fullness as well as desire for sweet and fatty foods were assessed using a visual analog scale. Allostatic load was calculated from physiologic markers. Aerobic fitness (VO_2_max), body composition (DXA), clinical labs, and quality-of-life questionnaires were also collected. Results: Participants with impaired fasting glucose (IFG) + impaired glucose tolerance (IGT) had a higher allostatic load, obesity, and insulin resistance compared with IFG or IGT (all *p* < 0.05), independent of fitness. IFG + IGT also had lower fasting ghrelin (*p* < 0.05) and no difference in fasting PYY. Hunger, fullness, and sweet ratings were comparable across groups, but fatty food ratings tended to be higher in IFG + IGT than NGT. Conclusions: Allostatic load was associated with altered fasting ghrelin levels in individuals with IFG + IGT, along with elevated body weight and insulin resistance. These findings suggest stress is a potential mechanism underlying appetite dysregulation in different forms of prediabetes.

## 1. Introduction

Nearly 98 million people in the U.S. have prediabetes [1]. This is problematic since people with prediabetes are at high risk of progressing to type 2 diabetes (T2D) and developing cardiovascular disease (CVD). Prediabetes is a broad term though because it characterizes individuals at high risk of developing T2D and/or CVD [2]. The American Diabetes Association defines prediabetes as elevated plasma glucose in the fasting and/or 120 min state after a 75 g glucose load [3]. This definition portrays, in turn, three phenotypes by which individuals may be diagnosed as hyperglycemic, i.e., impaired fasting glucose (IFG), impaired glucose tolerance (IGT), or both (IFG + IGT). While all phenotypes may present with obesity, it has been described that individuals with IGT often have reduced muscle insulin sensitivity, whereas individuals with IFG have low hepatic insulin sensitivity, such that studies show that IFG, IGT, and IFG + IGT are unique forms of glucose intolerance that promote CVD risk [4,5,6]. This raises questions about which etiological factors play roles that may impact appetite-mediated weight regulation mechanisms among these prediabetes phenotypes.

Stress is a multifaceted process integrating psychological, behavioral, and/or physiological pathways to elicit adaptation [7,8]. Chronic exposure to stress is often referred to as allostatic load and is believed to result in the “wear and tear” of biological systems that, in time, weakens stress-adaptive processes and reduces tissue resilience, thereby increasing disease risk [9]. Consumption of excess calories, fat, and/or sugar-laden foods is recognized as an environmental factor, leading to obesity risk as well as inflammation that can accentuate hypothalamic–pituitary–adrenal (HPA) axis activity (e.g., increased cortisol) [10]. In turn, elevated HPA axis activity has been linked to impaired appetite regulation [11,12]. However, whether prediabetes phenotypes have altered appetite regulation in relation to allostatic load compared with normal glucose-tolerant control (NGT) individuals is unknown. This is potentially relevant as insulin is an important hormone in appetite regulation [13], and people with IFG + IGT may have distorted hormonal responses to nutrient intake relative to their IFG or IGT counterparts [14,15,16]. Moreover, the altered insulin response may be potentially driven, in part, by elevations in acylated ghrelin and reductions in satiety-related hormones (e.g., PYY; protein tyrosine). To date, though, no study has examined appetite perception and/or hormones (e.g., ghrelin, PYY, and insulin) in people with excess body weight who have IFG, IGT, or IFG + IGT to better understand feeding behaviors across different pathologies. Therefore, we tested the hypothesis that IFG + IGT individuals would be characterized by less favorable appetite perceptions and hormones than those with IFG or IGT as well as NGT individuals, and this appetite dysregulation would relate to allostatic load.

## 2. Materials and Methods

### 2.1. Participants

Middle-aged to older adults (Table 1) who have prediabetes as defined by the American Diabetes Association criteria using a 75 g oral glucose tolerance test (OGTT) were involved in this cross-sectional study [3]. IFG was defined as having fasting glucose levels of 100–125 mg/dL but normal 120 min values of < 140 mg/dL. IGT was defined as having normal fasting glucose levels <100 mg/dL but elevated 120 min values of 140–199 mg/dL. IFG + IGT was defined as having high fasting and 120 min values. Participants were recruited from local communities using social media and/or newspaper flyers. Participants were not dieting or restricting food intake (e.g., low-carbohydrate diets, breakfast skippers, etc.), physically inactive (≤60 min/week of structured exercise), free of chronic disease (e.g., eating disorder, cancer, renal, cardiovascular, or any metabolic disease), non-smoking, and not using medication affecting insulin sensitivity (e.g., metformin, GLP-1 agonists) or vascular function (e.g., α-blockers). Clinical biochemistry assays, a 120 min 75 g oral glucose tolerance test, and a resting/exercise electrocardiogram were conducted to confirm eligibility, followed by a physical exam to ensure participant safety. The Epworth Sleepiness Scale and Pittsburgh Sleep Quality Index (PSQI) were also provided to characterize the likelihood of self-reported dozing or falling asleep during specific daily events (i.e., watching TV, sitting inactive, sitting in a car while stopped, etc.) as well as sleep across a one-month time span, as we did before [17]. This study is part of a larger clinical trial (Registration # NCT03355469) in adults with metabolic syndrome risk according to ATP III criteria and/or Framingham risk scores [18]. This study followed the Declaration of Helsinki standards, and all participants provided verbal and written consent prior to engagement in study protocols. The study was approved by the Institutional Review Board (IRB #19364 and #Pro2020002029).

### 2.2. Body Composition

Body mass was assessed on a digital scale with participants wearing minimal clothing. Height was also assessed with a stadiometer to calculate body mass index (BMI). Fat mass and lean body mass (LBM) were assessed via dual-energy X-ray absorptiometry (Lunar iDXA GE Medical Technologies, Madison, WI, USA). Waist circumference (WC) was assessed using a tape measure 2 cm above the umbilicus and averaged.

### 2.3. Cardiorespiratory Fitness

A maximal oxygen consumption (VO_2_max) test on a treadmill with indirect calorimetry (CareFusion, *V*_max_ CART, Yorba Linda, CA, USA, or Cosmed Quark, Chicago, IL, USA) was used to test cardiorespiratory fitness as described before [19]. Participants underwent a warm-up marked as the first 2 min of exercise, where a self-selected speed was chosen, which was then held constant for the duration of the test. The incline was raised every 2 min by 2.5% until VO_2_max was achieved.

### 2.4. Metabolic Control

After an overnight fast, participants reported to the Clinical Research Center (CRC) for resting metabolic rate (RMR) measurements via indirect calorimetry. In the supine position, individuals rested for 20 min and respiratory gases were measured for 15 min using a ventilated hood. The last 5 min were averaged to estimate RMR, which was multiplied by an activity factor of 1.2 to determine food intake needs (i.e., 55% carbohydrates, 15% protein, and 30% fat, with <10% from saturated fat). This diet was then provided 24 h prior to appetite regulation measures. Participants were also instructed to refrain from consumption of alcohol, caffeine, medications, and engagement in strenuous physical activity 24 h prior to the study visits.

### 2.5. Appetite Testing

Individuals reported to the CRC after an approximate 10 h overnight fast in the morning. Participants were asked to rest in a semi-supine position in a temperature-controlled room (22–23 °C). Appetite perception was tested via a 100 mm visual analog scale (VAS) [20]. Individuals were instructed to mark a single vertical line indicating their perceived feelings. The VAS was used to test hunger and fullness as well as desire for sweet and fatty foods. Then an intravenous catheter was placed in the antecubital fossa, dorsal hand, or forearm vein for glucose, insulin, PYY, and acylated ghrelin. A 75 g OGTT was then implemented, and at 30 min intervals after nutrient ingestion, VAS and blood samples were collected up to 120 min. Incremental area under the curve (iAUC) was calculated.

### 2.6. Food Intake

Dietary intake was assessed using 3-day food logs (i.e., 2 weekdays and 1 weekend day). Diet logs were analyzed using ESHA’s Food Processor Software (Version 11.1, Salem, OR, USA) to assess caloric and macronutrient intake.

### 2.7. Allostatic Load and General Health

Allostatic load was calculated using nine markers: SBP, DBP, BMI, WC, HDL, total cholesterol, hsCRP, HbA1c, and albumin, as they were used by prior work in adults and adolescents based on data availability [21]. One point was assigned for each biomarker ≥ 75th percentile of the sample, which was considered high-risk, except for albumin and HDL, where values ≤ 25th percentile were considered high-risk. Sex-specific cutoffs were applied for WC and HDL. All biomarkers were weighted equally, and the allostatic load score was the sum of points across the included biomarkers, with higher scores indicating greater physiological dysregulation. We also used the Veteran Rand General Health questionnaire to estimate individual perception of general health, emotional well-being, energy, and fatigue, as well as physical function.

### 2.8. Biochemical Analysis

Plasma glucose was collected in lithium heparin tubes and analyzed using the YSI 2300 StatPlus Glucose Analyzer system (Yellow Springs, OH, USA). Clinical labs (e.g., serum HDL, total cholesterol, etc.) were analyzed by assays (the University of Virginia’s Health System Laboratories or LabCorp). Remaining blood samples were collected in 3 mL EDTA vacutainers. Acylated ghrelin samples contained aprotinin, DPP-IV, and AEBSF (EMD Millipore, Billerica, MA, USA). PYY contained aprotinin and DPPIV, while insulin and hsCRP contained aprotinin only. Blood was centrifuged at 4 °C for 10 min at 3000 RPM. After centrifugation, HCl was immediately added to the aliquoted ghrelin plasma for acidification purposes. All blood was frozen at −80 °C until subsequent analysis and run in duplicate. Participant samples were analyzed in the same assay to minimize temporal variation. Acylated ghrelin, PYY, insulin, and hsCRP were determined using ELISA (EMD Millipore, Billerica, MA, USA, ALPCO, Salem, NH, USA, or R&D Systems, INC, Minneapolis, MN, USA, respectively).

### 2.9. Statistics

Data were analyzed using the software R (v. 4.4.1). Non-normally distributed data, as determined via QQ plots and Shapiro–Wilk, were log- or cube root-transformed for analyses. Data were analyzed via one-way ANOVA, and Tukey’s HSD post hoc analysis was performed when statistical differences between groups were observed. Effect sizes were calculated to assess the physiological relevance among group differences, with partial eta squared for one-way ANOVA analysis interpreted as small η^2^ = 0.01, medium η^2^ = 0.06, and large η^2^ = 0.14. Associations between allostatic load and appetite, hormones, and demographics were investigated using Spearman’s Rho. Significance was set at *p* ≤ 0.05. Data are expressed as mean ± SD.

## 3. Results

### 3.1. Participant Characteristics and Allostatic Load

**Figure 1:**
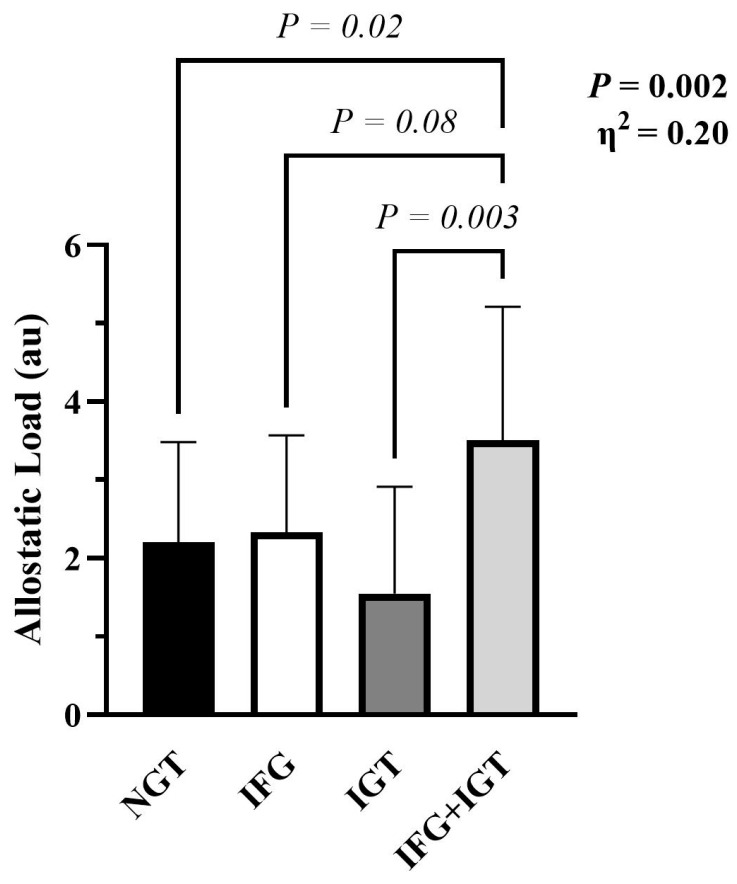
Allostatic load across prediabetes phenotypes. Data are mean ± SD. One-way ANOVA followed by Tukey’s HSD Test was used to identify group differences. Allostatic load was depicted in NGT (n = 20), IFG (n = 15), IGT (n = 11), and IFG + IGT (n = 22). ANOVA main effect *p*-values reported with Eta squared (η^2^).

Age and fitness were comparable between prediabetes phenotypes (Table 1). However, IFG + IGT had higher BMIs and waist circumference than IFG, IGT, or NGT (Table 1). When scaled to body weight, IFG + IGT had lower resting energy expenditure compared to IGT (Table 1). People with IFG + IGT also had higher allostatic load (*p* = 0.002, η^2^ = 0.20, Figure 1) despite no differences in general health, emotional well-being, and physical function (Table 2).

### 3.2. Appetite Perception and Habitual Dietary Intake

**Figure 2:**
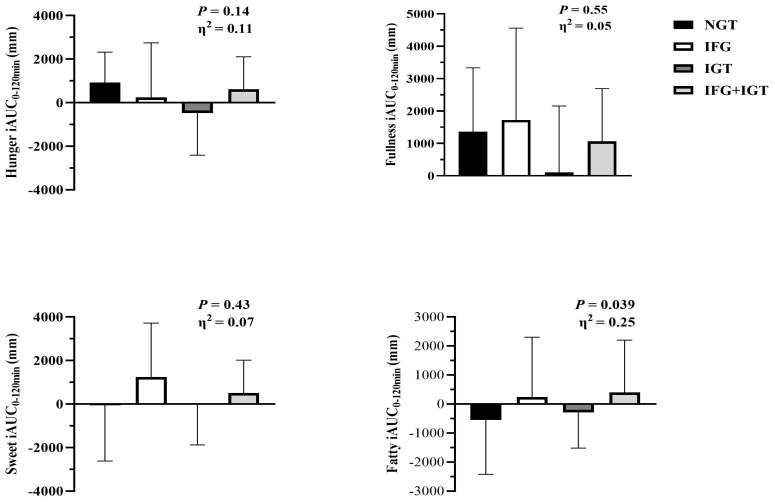
Appetite perception responses to glucose ingestion across prediabetes phenotypes. Data are mean ± SD. iAUC = incremental area under the curve (iAUC) for 120 min 75 g oral glucose tolerance test (OGTT). One-way ANOVA was used to assess group differences, and eta squared (η^2^) was used to test effect sizes.

There was no difference in fasting (Table 3) or post-prandial perception of hunger or fullness between prediabetes phenotypes (Figure 2). There was also no difference in desire for sweetness, although there was a statistical difference across phenotypes for desire for fatty foods in total phase iAUC (*p* = 0.039, η^2^ = 0.25), such that IFG + IGT tended to differ from NGT (*p* = 0.06, Figure 2). Total energy intake, along with carbohydrate, protein, and fat intake, were similar across groups (Table 3).

### 3.3. Glucose and Hormones

**Figure 3:**
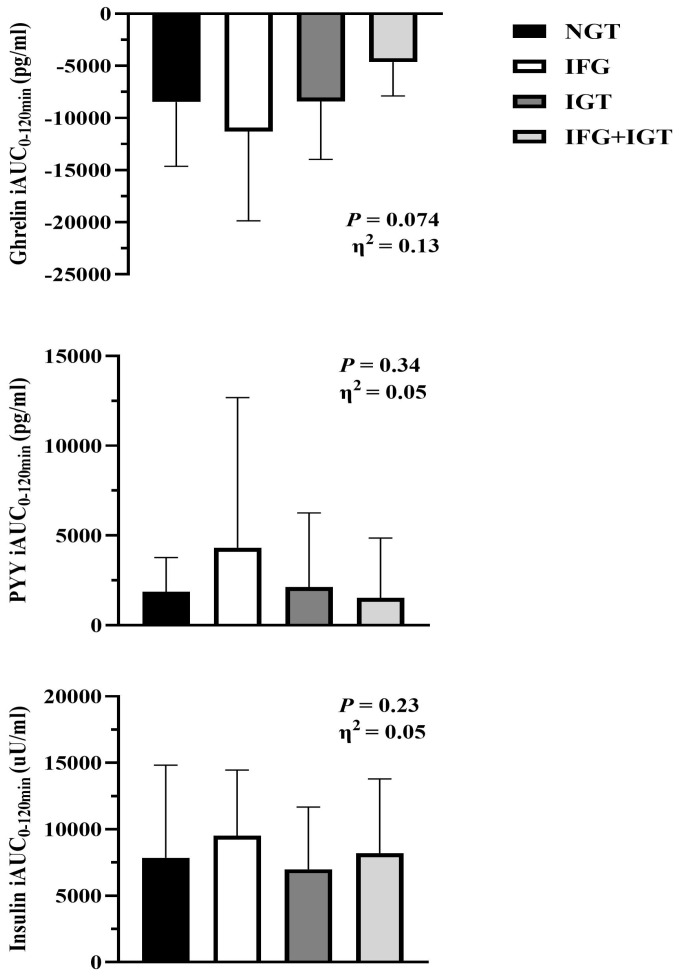
Ghrelin, PYY, and insulin responses to glucose ingestion across prediabetes phenotypes. Data are mean ± SD. iAUC = incremental area under the curve (iAUC) for 120 min of the 75 g oral glucose tolerance test (OGTT). One-way ANOVA followed by Tukey’s HSD Test was used to identify group differences. ANOVA main effect *p*-values are reported with Eta squared (η^2^), which was used to examine effect sizes.

As expected, fasting and 120 min plasma glucose were higher in IFG + IGT than other groups (Table 1). In turn, insulin levels were similarly elevated. Although HOMA-IR was higher in IFG + IGT versus other groups, there was no difference in the simple index of insulin sensitivity (Table 1). There was also no statistical difference in fasting leptin or PYY between NGT and prediabetes phenotypes (Table 3), nor was there an effect on post-prandial PYY iAUC responses during the OGTT (Figure 3). Fasting acylated ghrelin was different (Table 3), specifically between NGT and IFG + IGT (*p* = 0.029), which was consistent with a modest effect size in total phase iAUC period (*p* = 0.074, η^2^ = 0.13, Figure 3).

### 3.4. Correlations

Higher allostatic load was associated with increased fasting insulin (ρ = 0.55, *p* < 0.001) and PYY (ρ = 0.35, *p* = 0.004), as well as lower resting energy expenditure scaled to body weight (ρ = −0.42, *p* < 0.001). There were no relationships between allostatic load and age, fitness, measure of appetite perception, or habitual diet.

## 4. Discussion

The main finding of this work is that people with IFG + IGT had higher allostatic load, body weight, and insulin resistance than their counterparts, and this coincided with higher allostatic loads. Interestingly, this was paralleled by lower acylated ghrelin during fasting and post-prandial states, independent of PYY and perceptions of hunger as well as fullness. This aligned with the altered desire for fatty foods in those with IFG + IGT compared with their prediabetes and NGT counterparts. Our results suggest that people with IFG + IGT have increased appetite dysregulation compared to people with IFG or IGT, which is parallel with chronic stress. This highlights and expands prior literature by showing that stress may contribute to the unique appetite hormone profiles across prediabetes phenotypes.

Stress has been noted to cause some individuals to increase their food intake, while in others, there is either no change or a reduction in food intake [22,23,24]. The variation in such responses could be due to the type of stimuli, such that mild stressors promote increased food intake compared to strong stimuli evoking less food intake [25]. In either case, a more consistent observation has been that stress drives individuals to consume higher-fat and/or sugar-laden foods [26,27], even in the absence of hunger or caloric needs [28]. Moreover, prior work suggests that individuals with higher BMIs show increased propensity for weight gain in response to chronic stress relative to people with low BMIs who experience similar stress [24]. This parallels other work reporting that lean individuals have low food cravings and energy intake in the absence of hunger in both rest and stress conditions, while those who are overweight have higher levels [29]. Interestingly, people with IFG + IGT in the current work had higher body weight and waist circumference measures than their counterparts, despite no reported differences in hunger. Although we did not identify desires for fatty foods, these body weight findings suggest that obesity may occur in the absence of hunger and be related to stress-inducing physiological appetite alterations.

People with obesity have been reported to have increased activation in brain reward regions (e.g., striatum, insula, and thalamus) during exposure to food cues and stress [30]. In this later work, insulin resistance was related to the activation of the striatum and insula among individuals with obesity but not lean individuals. This would align with others reporting that high circulating insulin and insulin resistance may impair motivation pathways, resulting in heightened stress and food-cue responses [30,31]. In the current study, individuals with IFG + IGT had higher fasting and 120 min insulin levels, which coincided with higher fasting insulin resistance. Insulin has the ability to cross the blood–brain barrier to act on various brain regions, such as the hypothalamus, that regulate appetite. This is physiologically relevant since chronic stress acts on the HPA axis and stimulates the release of corticotropin-releasing factor (CRF) from the paraventricular nucleus of the hypothalamus. This general stress response is a normal adaptive mechanism to raise blood pressure, cardiac delivery, blood flow, as well as metabolism to support coping [32]. Importantly, leptin, insulin, and ghrelin act on the hypothalamus as well and can modulate CRF and adrenocorticotropic hormone (ACTH). In fact, insulin acts to suppress hunger in part via dampening ACTH release from the anterior pituitary gland, which triggers production of glucocorticoids (e.g., cortisol) in the adrenal cortex [33]. However, under chronic stress states, it is noteworthy that the presence of insulin with high glucocorticoid levels can increase abdominal fat [34], which is consistent with our work. Nevertheless, in line with our higher insulin results, participants with IFG + IGT had lower acylated ghrelin. This finding aligns with the prior literature [35], demonstrating a complex interaction of hormones being altered during stress. While insulin and ghrelin appear to maintain their normal interactions, it remains of interest that higher insulin levels were accompanied by similar hunger responses. This could suggest that, on a neutral level, the brain was somewhat insulin resistant, requiring greater signaling to elicit similar hunger and fullness responses. Further work is necessary to discern the role of stress on appetite perception and hormones, given prior reports in some [36,37], but not all [38], past work we performed, suggesting IFG + IGT remain more insulin resistant and glucose intolerant following exercise training than those with IGT or IFG.

Chronic stress is often related to anxiety and depression, and it has been postulated that overconsumption of food may act to comfort individuals [39,40]. Prior work also suggests people with prediabetes may have heightened psychosocial problems before type 2 diabetes onset [41]. If people with IFG + IGT in our study reported altered well-being, it would then be reasonable to suspect that emotion-related pathways contribute to the possibility of appetite dysregulation. However, no differences in emotional health were observed in this cohort of participants, which suggests other factors likely explain the differences in appetite regulation. Another factor to consider impacting appetite hormones in this study is sleep. A lack of sleep is known to increase the risk of obesity [34], insulin resistance [42], and ghrelin [43], as well as lower leptin levels [43], although the influence on plasma cortisol is mixed [44,45]. Regardless, individuals in our study reported no difference in subjective sleep duration, nor did they indicate differences in drowsiness throughout the day. As a result, habitual sleep is not likely to explain the differences in hormonal appetite regulation across these prediabetes phenotypes.

Appetite regulation is an integrative process of biological mechanisms that modulate the need for energy in combination with hedonic processes (i.e., wanting) that modulate food intake [46]. Tonic processes in appetite control typically reflect stable or slow-changing mechanisms, whereas episodic eating behavior occurs within or between a given meal. In the present work, the hormonal shifts observed with insulin and ghrelin reflect episodic shifts. However, it is interesting to note that we observed no differences in resting metabolic rate, which is a primary tonic regulator of feeding behavior [46]. People with IFG + IGT were heavier on average, and it would have been expected that the higher BMI would correspond with higher resting energy expenditures. Whether stress suppressed this resting energy expenditure in IFG + IGT and created somewhat of a constrained energy system is unclear, as there are mixed results on the impact stress has on resting metabolism [47]. In fact, our results suggest that a lower resting metabolic rate scaled to body mass related to elevated allostatic load. In turn, further work is warranted as the lower relative resting metabolic rate in these people with IFG + IGT may have contributed to lower hunger scores and promoted the null effects despite being heavier.

There are limitations to the present work that might impact our findings. We cannot generalize these findings from an OGTT to mixed meals across the day. However, the hormonal response is similar such that differences in the direction of hormonal change is unlikely relative to mixed meals [48,49]. We recognize that use of self-reported food logs is susceptible to under-/over-reporting of food intake, and this could affect our interpretation of energy intake between groups. We did not directly measure cortisol or catecholamines in this study to assess stress, although there were no noted differences in general perceived well-being. Future work should consider the collection of urinary or salivary measures of hormones throughout the day to fully understand how stress influences appetite-related hormonal responses. *C*-peptides were not assessed to assess insulin secretion. As a result, our indices of insulin resistance may over- or under-estimate insulin resistance calculations performed in the present work. Moreover, consideration of social determinants of health (e.g., marital status, employment, etc.) ought to be considered. Lastly, there is no consensus on biomarkers and/or measurement approach for allostatic load calculations, and additional work is needed to identify optimal equations for this physiological outcome [50].

## 5. Conclusions

People with IFG + IGT had a higher allostatic load, body weight, and degree of insulin resistance than those with IFG or IGT alone. People with IFG + IGT, in turn, also had lower acylated ghrelin levels even though they had comparable hunger scores. These findings suggest people with IFG + IGT may have an increased risk of appetite dysregulation compared to their counterparts. Thus, these findings point towards biological stress as a potential factor modulating appetite regulation in people with obesity and hyperglycemia. Additional attention to such issues may enable tailored treatments to improve appetite responses and combat obesity-mediated chronic disease risk.

## Figures and Tables

**Table 1:** Participant characteristics.

	NGT	IFG	IGT	IFG + IGT	*p*	η^2^
N	28	20	18	32		
F/M	23/5	14/6	16/2	24/8		
Demographics						
Age (y)	52.9 ± 7.6	56.6 ± 6.3	55.4 ± 8.4	57.0 ± 7.4	0.17	0.05
VO_2_max (mL/kg/min) *	21.9 ± 4.5	23.9 ± 4.2	22.1 ± 3.0	22.2 ± 5.3	0.38	0.03
ATP III Criteria	3.07 ± 0.86	3.45 ± 0.83	2.72 ± 0.75	3.59 ± 0.76	0.002	0.15
Weight (kg) *	101 ± 21.4	99.8 ± 17.1	88.8 ± 18.3	105 ± 21.7 ^	0.037	0.09
Body Fat (%)	44.9 ± 5.28	43.8 ± 5.71	44.7 ± 5.51	44.2 ± 6.55	0.93	<0.01
Lean Mass (kg)	51.7 ± 11.4	53.5 ± 9.36	45.3 ± 8.11	54.7 ± 10.7 ^	0.023	0.12
RMR (kcal/d)	1402 ± 193	1366 ± 426	1434 ± 250	1426 ± 262	0.53	0.03
RMR (kcal/kg/d)	14.4 ± 3.12	14.3 ± 2.87	16.7 ± 3.57	13.9 ± 2.78 ^	0.049	0.09
AL Parameters						
SBP (mmHg)	132 ± 14.2	129 ± 10.7	129 ± 7.94	133 ± 14.7	0.59	0.02
DBP (mmHg)	80.1 ± 9.91	78.2 ± 8.76	80.7 ± 6.30	79.8 ± 9.01	0.84	<0.01
BMI (kg/m^2^) *	35.2 ± 5.4	34.8 ± 4.4	32.6 ± 5.7	36.5 ± 5.7	0.079	0.07
WC (cm) *	110 ± 14.3	112 ± 13.4	102 ± 11.9	114 ± 11.7 ^	0.022	0.10
HDL (mg/dL) *	51.0 ± 11.6	49.0 ± 10.3	52.1 ± 14.7	46.9 ± 10.3	0.43	0.03
TC (mg/dL)	212 ± 46.9	210 ± 31.1	187 ± 48.5	201 ± 37.8	0.23	0.04
hsCRP *	5.52 ± 5.16	3.92 ± 3.80	3.35 ± 3.69	6.18 ± 6.39	0.18	0.07
HbA1c (%) *	5.36 ± 0.23	5.57 ± 0.31	5.57 ± 0.36	5.98 ± 0.48 †‡^	<0.001	0.32
Albumin (g/dL)	4.32 ± 0.29	4.37 ± 0.34	4.36 ± 0.29	4.22 ± 0.26	0.21	0.05
OGTT						
Fasting Glc (mg/dL) *	92.9 ± 7.2	108 ± 6.9†^	90.0 ± 5.7	117 ± 13.0 †‡^	<0.001	0.63
120 min Glc (mg/dL) *	114 ± 13.4	116 ± 15.1	164 ± 17.6 †‡	177 ± 28.0 †‡	<0.001	0.71
Glc iAUC_0–120min_ (mg/dL) *	4650 ± 1807	4394 ± 1827	7981 ± 2156 †‡	8044 ± 2497 †‡	<0.001	0.39
Fasting Insulin (μU/mL) *	10.7 ± 6.08	14.0 ± 9.24	9.49 ± 5.28	17.6 ± 11.1 †^	0.013	0.12
120 min Insulin (μU/mL) *	61.8 ± 56.9	74.0 ± 41.8	97.3 ± 109	150 ± 155 †‡	<0.001	0.19
SI_IS_ (au) *	0.30 ± 0.13	0.30 ± 0.13	0.31 ± 0.13	0.30 ± 0.14	0.98	<0.01
HOMA-IR (au) *	2.53 ± 1.5	3.74 ± 2.6	2.14 ± 1.2	5.21 ± 3.5 †^	<0.001	0.21

Data are mean ± SD. BMI = body mass index. VO_2_max = aerobic capacity relative to mean body weight (mL/kg/min). RMR = resting metabolic rate. ATP III = adult treatment panel III. AL = allostatic load. SBP = systolic blood pressure. DBP = diastolic blood pressure. WC = waist circumference. HDL = high-density lipoprotein. TC = total cholesterol. hsCRP = high-sensitivity *C*-reactive protein. HbA1c = hemoglobin A1c. Glc = glucose. iAUC = incremental area under the curve. SI_IS_ = simple index of insulin sensitivity. HOMA-IR = homeostatic model assessment of insulin resistance. * Log-transformed for analysis. One-way ANOVA followed by Tukey’s HSD Test was used to identify group differences. † Significant pairwise comparison compared to NGT. ‡ Significant pairwise comparison compared to IFG. ^ Significant pairwise comparison compared to IGT. Eta squared (η^2^) was used to examine effect sizes.

**Table 2:** Perceived health and sleep.

	NGT	IFG	IGT	IFG + IGT	*p*	η^2^
Perceived Health						
General Health (au)	61.6 ± 16.1	66.8 ± 19.5	66.1 ± 19.3	58.4 ± 22.6	0.55	0.03
Energy and Fatigue (au)	45.6 ± 14.6	55.6 ± 18.7	57.5 ± 14.8	45.2 ± 24.4	0.13	0.08
Emotional Well-Being (au)	74.7 ± 9.3	75.8 ± 16.5	74.7 ± 16.8	79.0 ± 15.3	0.76	0.02
Physical Function	81.4 ± 15.1	82.8 ± 15.3	84.6 ± 16.0	79.5 ± 15.0	0.81	0.01
Sleeping Habits						
PSQI (au)	7.58 ± 3.02	6.47 ± 3.45	6.57 ± 3.41	6.05 ± 3.36	0.52	0.03
Epworth (au)	5.56 ± 4.16	5.25 ± 3.63	5.47 ± 4.06	7.52 ± 4.92	0.19	0.05

Data are mean ± SD. One-way ANOVA was used to assess group differences, and eta squared (η^2^) was used to test effect sizes. Perceived health questionnaire group breakdown included: NGT (n = 21), IFG (n = 16), IGT (n = 12), and IFG + IGT (n = 20).

**Table 3:** Fasting appetite, hormones, and food intake.

	NGT	IFG	IGT	IFG + IGT	*p*	η^2^
Fasting Appetite						
Hunger (mm)	31.6 ± 21.5	35.9 ± 26.0	38.3 ± 25.7	36.6 ± 26.1	0.84	0.01
Fullness (mm)	23.1 ± 19.5	19.3 ± 23.2	35.9 ± 31.4	24.1 ± 25.2	0.99	<0.01
Sweet (mm)	64.7 ± 27.7	67.9 ± 26.3	64.2 ± 26.3	69.8 ± 28.5	0.96	<0.01
Fatty (mm)	60.0 ± 28.3	59.9 ± 25.6	66.9 ± 24.0	55.9 ± 31.5	0.55	0.03
Fasting Hormones						
Leptin (ng/mL) *	50.1 ± 21.9	42.4 ± 24.7	43.6 ± 30.8	53.4 ± 31.3	0.70	0.02
Ghrelin (pg/mL) *	190 ± 106	203 ± 137	180 ± 90.3	112 ± 67.3 †	0.013	0.15
PYY (pg/mL) *	105 ± 71.7	101 ± 52.5	75.1 ± 41.4	106 ± 56.7	0.61	0.02
Food Intake						
Total (kcals)	1901 ± 396	1975 ± 556	2057 ± 686	1948 ± 504	0.93	<0.01
Fat (g)	88.8 ± 30.4	86.7 ± 36.2	85.5 ± 28.1	80.9 ± 30.1	0.91	<0.01
CHO (g)	193 ± 40.4	201 ± 78.0	238 ± 40.4	212 ± 65.6	0.53	0.04
Soluble Fiber (g)	0.96 ± 0.98	1.24 ±0.85	0.64 ± 0.79	0.81 ± 0.71	0.11	0.11
Protein (g)	82.3 ± 16.3	90.7 ± 32.6	86.8 ± 26.8	91.7 ± 46.7	0.98	<0.01

Data are mean ± SD. One-way ANOVA followed by Tukey’s HSD Test was used to identify group differences. † Significant pairwise comparison compared to NGT. Eta squared (η^2^) was used to examine effect sizes. Ghrelin results included: NGT (n = 22), IFG (n = 12), IGT (n = 12), IFG + IGT (n = 25).

## Data Availability

These data have not been made publicly available due to ethical reasons. However, the corresponding author (S.K.M.) can provide further information on the data upon reasonable request.
